# Safety and tolerability of fremanezumab in patients with episodic and chronic migraine: a pooled analysis of phase 3 studies

**DOI:** 10.1177/03331024221076485

**Published:** 2022-03-25

**Authors:** Hans Christoph Diener, Peter McAllister, Tim P Jürgens, Yoel Kessler, Xiaoping Ning, Joshua M Cohen, Verena Ramirez Campos, Steve Barash, Stephen D Silberstein

**Affiliations:** 1 Institute for Medical Informatics, Biometry, and Epidemiology, Medical Faculty of the University Duisburg-Essen, Essen, Germany; 2 New England Institute for Neurology and Headache, Stamford, CT, USA; 3 Headache Center North-East, Department of Neurology, University Medical Center Rostock, Rostock, Germany; 4 Teva Branded Pharmaceutical Products R&D, Inc., West Chester, PA, USA; 5 Jefferson Headache Center, Philadelphia, PA, USA

**Keywords:** fremanezumab, calcitonin gene-related peptide, safety, cardiovascular

## Abstract

**Background:**

Fremanezumab, a fully humanized monoclonal antibody that selectively targets calcitonin gene-related peptide, has demonstrated efficacy for preventive treatment of episodic and chronic migraine. Since calcitonin gene-related peptide is expressed within the cardio- and cerebrovascular system and may have cardioprotective effects, it is critical to understand the cardio- and cerebrovascular safety of fremanezumab.

**Methods:**

This was a pooled analysis of three randomized, double-blind, placebo-controlled, phase 3, 12-week trials in which patients with episodic migraine or chronic migraine received quarterly fremanezumab, monthly fremanezumab, or placebo. Incidences of overall and serious adverse events were analyzed. Cardio- and cerebrovascular adverse events (CVAEs) were analyzed in subgroups stratified by cardio- and cerebrovascular medical history, cardiovascular risk factors (CVRFs), and use of cardio- and cerebrovascular medications or triptans.

**Results:**

Two thousand, eight hundred and forty-two patients were included in the study. Overall (58–65%) and serious adverse events (<1–2%) occurred in similar proportions across fremanezumab and placebo groups. CVAEs were infrequent, regardless of cardio- and cerebrovascular medical history (2–6%). CVAEs occurred in low, similar proportions of patients with CVRFs and those using cardio- and cerebrovascular medications or triptans. No cardio- and cerebrovascular signals were identified.

**Conclusion:**

Fremanezumab demonstrated a favorable overall and cardio- and cerebrovascular safety profile in more than 2800 patients with episodic migraine or chronic migraine, regardless of cardio- and cerebrovascular medical history, CVRFs, or medication use.

**Trial Registrations:** NCT02629861 (HALO EM, https://clinicaltrials.gov/ct2/show/NCT02629861), NCT02621931 (HALO CM, https://clinicaltrials.gov/ct2/show/NCT02621931), NCT03308968 (FOCUS, https://clinicaltrials.gov/ct2/ show/NCT03308968)

## Introduction

Migraine is a complex and burdensome disease that affects >1 billion individuals and is the second leading cause of years lived with disability worldwide (1). Episodic migraine (EM), and especially chronic migraine (CM), have a substantial negative effect on health-related quality of life for patients (2). Preventive medications are recommended in patients with frequent or disabling attacks to reduce the frequency of migraine attacks and/or to mitigate their impact. While there are numerous options available for migraine preventive therapy, such as beta-blockers, antihypertensives, anticonvulsants, antidepressants, and onabotulinumtoxinA, these medications were not developed specifically for migraine (3). Among these therapies, only onabotulinumtoxinA is approved for the preventive treatment of CM (4). Due in part to suboptimal tolerability, efficacy, and adherence to these medications, there was an unmet need for migraine-specific preventive treatments with proven tolerability, safety, and efficacy in the reduction of both frequency, severity, and duration of migraine attacks (5).

The development of monoclonal antibodies (mAbs) that target the calcitonin gene-related peptide (CGRP) pathway has significantly advanced migraine preventive therapy. Four CGRP pathway–targeting mAbs (fremanezumab, erenumab, galcanezumab, and eptinezumab) are approved for the preventive treatment of EM and CM (6). Fremanezumab, galcanezumab, and eptinezumab target the CGRP ligand, while erenumab targets the CGRP receptor. CGRP is a neuropeptide that is produced in peripheral sensory neurons and the central nervous system and is integral to the pathophysiology of migraine (7). Blockade of the CGRP pathway has not only been found to be effective in migraine prevention, but also in aborting migraine attacks (6).

In addition to the pial and dural vasculature, CGRP receptors are expressed within the cardio- and cerebrovascular (CV) system and are localized in sensory nerve fibers and around the peripheral arteries of the heart (8,9). CGRP is a potent vasodilator that has a role in arterial function and has been hypothesized to regulate local vascular responses within the CV system, including mediating collateral recruitment during cerebral ischemia (8,10). Because of the potential cardioprotective capacity of CGRP, understanding the CV safety of its inhibition by CGRP pathway–targeting mAbs is of critical importance (8).

Triptans, a first-line option for the acute treatment of migraine attacks, are selective 5-hydroxytriptamine (5-HT_1B/1D_) receptor agonists. 5-HT_1B/1D_ receptors are expressed in CV and coronary blood vessels, and the stimulation of the 5-HT_1B_ receptor results in minor cranial vasoconstriction (11). Because of their vasoconstrictive capacity, triptans can theoretically increase the risk of serious ischemic events and have therefore been contraindicated in patients with significant CV disease (11–13). However, this recommendation was based on the incorrect assumption that most acute coronary syndromes and ischemic strokes are caused by vasoconstriction, and a study by Goldstein et al. showed that even high doses of triptans did not significantly constrict coronary artery diameter (14). Furthermore, a retrospective cohort study of 130,411 patients with migraine and 130,411 matched controls showed no increase in the risk of myocardial infarction with current or recent triptan use, supporting the CV safety of triptans (15). Nevertheless, given that triptans are used for acute migraine treatment alongside preventive medications, including CGRP pathway–targeting mAbs, and that each of these drugs has a potential role in CV function, it is important to confirm the CV safety of their concomitant use.

Fremanezumab is a fully humanized mAb (IgG2Δa) that selectively targets CGRP and is approved for the preventive treatment of migraine in adults (16). It has demonstrated good safety, tolerability, and efficacy for the preventive treatment of migraine in patients with EM or CM, including those with difficult-to-treat migraine, in three randomized, double-blind, placebo-controlled, phase 3 trials (17–19). The long-term safety and efficacy of fremanezumab treatment have been demonstrated for up to 12 months in patients with EM or CM in a randomized, double-blind, parallel-group, phase 3 study (20). Here, we present the overall and CV safety of fremanezumab over 12 weeks of the placebo-controlled period from three phase 3 trials in patients with EM or CM with or without CV medical history, with CV risk factors (CVRFs), and with concomitant use of medications for the treatment of CV disease or the intake of triptans.

## Methods

### Study design and patients

This pooled analysis included safety and tolerability data from three randomized, double-blind, placebo-controlled clinical trials (ClinicalTrials.gov Identifiers: NCT02629861 [HALO EM], NCT02621931 [HALO CM], and NCT03308968 [FOCUS]; Table 1) (17–19) that evaluated the safety and efficacy of fremanezumab for the preventive treatment of migraine. The trials comprised a screening visit, a 28-day preintervention period, and a 12-week treatment period. The HALO studies concluded with a final evaluation at 12 weeks, though most patients rolled over to the 1-year extension trial, while the FOCUS study included a subsequent 12-week open-label period. Patients in all studies had a follow-up visit 6 months after the last dose of fremanezumab. Data presented here are from the 12-week, double-blind, placebo-controlled treatment period only. The study protocols and primary results for these studies have been published previously (17–19).

**Table 1.** Clinical studies in the pooled safety analysis.

Study	Patient population	Study design	n	Treatment groups	Primary outcome
NCT02629861 (HALO EM) (17)	EM	Randomized, double-blind, placebo-controlled,phase 3 trial	875	Fremanezumab monthly: 225/225/225 mg(n = 290)Fremanezumab quarterly: 675 mg/PBO/PBO(n = 291) Placebo: PBO/PBO/PBO(n = 294)	Mean change from baseline in the monthly average number of migraine days during the 12-week treatment period
NCT02621931 (HALO CM) (18)	CM	Randomized, double-blind, placebo-controlled,phase 3 trial	1130	Fremanezumab monthly: 675/225/225 mg(n = 376)Fremanezumab quarterly: 625 mg/PBO/PBO(n = 379)Placebo: PBO/PBO/PBO(n = 375)	Mean change from baseline in the monthly average number of headache days of at least moderate severity during the 12-week treatment period
NCT03308968 (FOCUS) (19)	EM or CM and prior inadequate response to 2–4 classes of migraine preventive medications	Randomized, double-blind, placebo-controlled,phase 3 trial	838	Fremanezumab quarterly: EM/CM: 625 mg/PBO/PBO(n = 276)Fremanezumab monthly: EM: 225/225/225 mgCM: 675/225/225 mg(n = 283)Placebo: PBO/PBO/PBO(n = 279)	Mean change in the monthly average number of migraine days during the 12-week treatment period

EM: episodic migraine; CM: chronic migraine; PBO: placebo.

Eligible participants for all the studies were 18 to 70 years of age, with a history of migraine with onset at or prior to age 50 years and for at least 12 months prior to screening. The HALO EM study included patients with EM (≥6 and <15 headache days per month, with ≥4 days fulfilling the International Classification of Headache Disorders 3 beta version [ICHD-3 beta] criteria (21) for migraine with aura or without aura, probable migraine, or use of triptans or ergots to treat an established headache). The HALO CM study included patients with CM (≥15 headache days per month, with ≥8 days fulfilling the ICHD-3 beta criteria for migraine with or without aura, probable migraine, or use of triptans or ergots to treat an established headache). The FOCUS study included patients with EM or CM and documented inadequate response (no clinically meaningful improvement per treating physician’s judgment after ≥3 months of therapy; treatment had to be interrupted because of adverse events (AEs) that made it intolerable for the patients, or the medication was contraindicated or unsuitable for the preventive treatment of migraine for the patient) to 2 to 4 classes of migraine preventive medications (beta-blockers, tricyclic antidepressants, calcium channel blockers, angiotensin II receptor antagonists, onabotulinumtoxinA, or valproic acid) within the past 10 years (19).

### Ethics approvals and patient consent

The studies included in this analysis were conducted in compliance with the International Conference for Harmonisation guidelines for Good Clinical Practice, the Declaration of Helsinki, and relevant national and local regulations. Patients from each study provided written informed consent before study procedures and assessments were conducted.

### Randomization and treatment procedures

Patients were randomly assigned 1:1:1 to quarterly fremanezumab (675 mg fremanezumab at baseline, matched monthly placebo at weeks 4 and 8), monthly fremanezumab (for patients with EM: 225 mg fremanezumab at baseline, week 4, and week 8; for patients with CM: 675 mg fremanezumab at baseline, 225 mg fremanezumab at weeks 4 and 8), or matched monthly placebo by subcutaneous (SC) injection. Randomization was performed using electronic interactive response technology, and patients were stratified according to baseline preventive medication use, sex, country, and migraine classification (for the FOCUS study). The sponsor, patients, investigators, and designated personnel were blinded to treatment assignments.

### Safety endpoints

Safety and tolerability endpoints included incidence of overall AEs, serious AEs, and AEs leading to discontinuation. The incidence of all cardio- or cerebrovascular AEs (CVAEs), including arrhythmias and electrocardiogram changes, was assessed in several patient subgroups: patients with and without CV medical history (i.e. CV disease-related medical conditions reported in the patient’s medical history); patients with CVRFs (i.e. potential risk factors for developing CV disease); patients who were receiving CV medication(s) at baseline; and patients who used triptans throughout the study. While patients with a history of serious CV disease, such as myocardial infarction or stroke, were excluded from this study, patients with CV medical history (Table S1) and CVRFs (Table S2) were included.

### Data analysis

The patient population presented here comprises the safety populations from each study, which consisted of all patients who received ≥1 dose of the study drug. Descriptive statistics are reported for continuous and categorical variables. For continuous variables, mean and standard deviation (SD) are reported. For categorical variables, count (n) and percentage of patients in each category are provided. No statistical analyses are reported in this study.

## Results

### Patient population

A total of 2842 patients were included in the pooled safety population. Demographic characteristics were similar across treatment groups (Table 2). Between 85% and 86% of patients in each treatment group were female, and the proportion of patients with EM ranged from 42% to 43% (Table 2). The most prevalent CV medical history and CVRFs among the study population are provided in Tables S1 and S2, respectively. The most commonly reported CV medical history was hypertension (quarterly fremanezumab, 8%; monthly fremanezumab, 8%; total fremanezumab, 8%; placebo, 9%; Table S1). Other CV medical history was reported in ≤1% of patients across treatment groups (Table S1). The most common CVRFs were obesity (22–25%), history of CV disease (15–16%), and hormonal birth control pill use (18–19%; Table S2).

**Table 2.** Baseline demographics and patient characteristics.

	Quarterly fremanezumabEM/CM: 675 mg/PBO/PBOn = 943	Monthly fremanezumabEM: 225/225/225 mgCM: 675/225/225 mgn = 954	Total fremanezumabn = 1897	PBOn = 945
Mean age (SD), years	42.8 (11.8)	43.0 (12.1)	42.9 (12.0)	42.9 (12.0)
Sex, female, n (%)	811 (86)	813 (85)	1624 (86)	808 (86)
Race, White, n (%)	787 (83)	804 (84)	1591 (84)	787 (83)
Mean BMI (SD), kg	26.3 (5.0)	26.0 (5.0)	26.2 (5.0)	26.4 (4.8)
Indications, n (%)				
EM	398 (42)	401 (42)	799 (42)	404 (43)
CV medical history	167 (18)	158 (17)	325 (17)	153 (16)
≥2 CVRFs	174 (18)	156 (16)	330 (17)	169 (18)
≥3 CVRFs	68 (7)	54 (6)	122 (6)	61 (6)
CV medication use at baseline	87 (9)	91 (10)	178 (9)	102 (11)
Concomitant triptan use	393 (42)	365 (38)	758 (40)	365 (39)

EM: episodic migraine; CM: chronic migraine; PBO: placebo; SD: standard deviation; BMI: body mass index; CV: cardio- and cerebrovascular; CVRF: cardiovascular risk factor.

### Overall safety and tolerability

The proportion of patients who experienced ≥1 AE was similar across treatment groups (quarterly fremanezumab, 65%; monthly fremanezumab, 62%; total fremanezumab, 63%; placebo, 58%; Table 3). Injection-site pain, injection-site induration, and injection-site erythema were the most commonly reported AEs and had similar incidences across treatment groups (Table 3). Apart from nausea (quarterly fremanezumab, 2%; monthly fremanezumab, 1%; total fremanezumab, 1%; placebo, 2%), gastrointestinal AEs were reported in <2% of patients in each of the fremanezumab treatment groups. Serious AEs and AEs leading to discontinuation from the study drug occurred in ≤2% of patients across treatment groups (Table S3). The most commonly reported AEs leading to discontinuation were also injection site related (erythema and rash); each of these led to discontinuation at low, similar rates across treatment groups (all groups, <1%). Two deaths occurred during the trials; one patient in HALO EM died by intentional overdose of diphenhydramine 109 days after receiving a single quarterly dose of fremanezumab and 38 days after withdrawing consent from the study, and one patient in HALO CM died of chronic obstructive pulmonary disease 69 days after receiving a single quarterly dose of fremanezumab. An autopsy was performed in both cases to confirm the cause of death; intake of the study drug was not considered causal for either death (17,18).

**Table 3.** Overall safety of fremanezumab in the pooled safety population.

AEs, n (%)	Quarterly fremanezumabEM/CM: 675 mg/PBO/PBOn = 943	Monthly fremanezumabEM: 225/225/225 mgCM: 675/225/225 mgn = 954	Total fremanezumabn = 1897	PBOn = 945
Most common AEs (≥2% incidence in any fremanezumab treatment group)				
Injection-site pain	211 (22)	195 (20)	406 (21)	188 (20)
Injection-site induration	146 (15)	175 (18)	321 (17)	125 (13)
Injection-site erythema	155 (16)	145 (15)	300 (16)	116 (12)
Injection-site hemorrhage	16 (2)	14 (1)	30 (2)	16 (2)
Injection-site pruritus	13 (1)	17 (2)	30 (2)	5 (<1)
Fatigue	18 (2)	16 (2)	34 (2)	12 (1)
Nasopharyngitis	43 (5)	33 (3)	76 (4)	41 (4)
Upper respiratory tract infection	33 (3)	42 (4)	75 (4)	33 (3)
Urinary tract infection	17 (2)	15 (2)	32 (2)	16 (2)
Influenza	10 (1)	15 (2)	25 (1)	9 (<1)
Nausea	15 (2)	12 (1)	27 (1)	23 (2)
Dizziness	14 (1)	18 (2)	32 (2)	13 (1)

AE: adverse event; EM: episodic migraine; CM: chronic migraine; PBO: placebo.

### CV safety in patients with CV medical history or risk factors

To evaluate the CV safety of fremanezumab with respect to relevant patient characteristics, we analyzed the incidence of CVAEs in patient subgroups according to CV medical history, CVRFs, and concomitant CV medication and triptan use. Among patients with CV medical history, the incidence of CVAEs ranged from 3% to 6% (Table 4). Hypertension was the most commonly reported CVAE among patients with CV medical history but did not occur in >2% of patients with CV medical history in any treatment group (quarterly fremanezumab, 2%; monthly fremanezumab, <1%; total fremanezumab, 1%; placebo, <1%; Table S4). No other CVAE occurred in >1% of patients with CV medical history. CVAEs occurred in low, similar proportions of patients without CV medical history (all treatment groups, 2%; Table S4). Similar to patients with CV medical history, hypertension, while rare, was the most frequently reported CVAE among patients without CV medical history (<1%, all treatment groups; Table S4).

**Table 4.** CVAEs in patients with CV medical history.

CVAEs, n (%)	Quarterly fremanezumabEM/CM: 675 mg/PBO/PBOn = 167	Monthly fremanezumabEM: 225/225/225 mgCM: 675/225/225 mgn = 158	Total fremanezumabn = 325	PBOn = 153
≥1 CVAE	6 (4)	10 (6)	16 (5)	4 (3)
AEs with an incidence of ≥1 patient in any treatment/dose group				
Hypertension	4 (2)	1 (<1)	5 (2)	1 (<1)
Palpitations	0	2 (1)	2 (<1)	1 (<1)
Atrial fibrillation	0	1 (<1)	1 (<1)	0
ECG QT prolongation	0	1 (<1)	1 (<1)	0
Increased heart rate	0	1 (<1)	1 (<1)	0
Supraventricular tachycardia	1 (<1)	0	1 (<1)	0
Bradycardia	0	0	0	1 (<1)
Tachycardia	0	0	0	1 (<1)
Increased blood pressure	0	1 (<1)	1 (<1)	0
Hot flush	1 (<1)	0	1 (<1)	0
Hypertensive crisis	0	1 (<1)	1 (<1)	0
Hypotension	0	1 (<1)	1 (<1)	0
Raynaud’s phenomenon	0	1 (<1)	1 (<1)	0

CVAE: cardio- and cerebrovascular adverse event; CV: cardio- and cerebrovascular; EM: episodic migraine; CM: chronic migraine; PBO: placebo;AE: adverse event; ECG: electrocardiogram.

CVAEs were infrequent in patients with CVRFs, occurring in ≤2% of patients with ≥2 or ≥3 CVRFs (Table 5). Hypertension (defined as an AE according to the investigator’s discretion, patient history, and appropriate guidelines) was the most frequently reported CVAE in patients with ≥2 or ≥3 CVRFs, but occurred at low, similar rates across treatment groups (≥2 CVRFs: quarterly fremanezumab, 2%; monthly fremanezumab, <1%; total fremanezumab, 1%; placebo, <1%; ≥3 CVRFs: quarterly fremanezumab, 3%; monthly fremanezumab, 2%; total fremanezumab, 2%; placebo, 2%; Table 5). For patients with both ≥2 CVRFs and CV medical history, the proportion of patients with ≥1 CVAE was between 3% and 8% (quarterly fremanezumab, 4%; monthly fremanezumab, 8%; total fremanezumab, 6%; placebo, 3%; Table S5). The incidence of individual CVAEs was infrequent in this subgroup; hypertension was the most commonly reported CVAE, occurring in 3% of the quarterly fremanezumab group, <1% of the monthly fremanezumab group, 2% of the total fremanezumab group, and <1% of the placebo group. CVAEs occurred at similar, low rates across treatment groups in patients with ≥2 CVRFs without CV medical history (quarterly fremanezumab, 2%; monthly fremanezumab, 4%; total fremanezumab, 3%; placebo, 4%; Table S5), and none of the CVAEs reported occurred in >1 patient in any individual treatment group. In patients with ≥4 CRVFs, no CVAEs were reported in any treatment group.

**Table 5.** CVAEs in patients with ≥2 and ≥3 CVRFs.

	Quarterly fremanezumab		Monthly fremanezumab		Total fremanezumab		PBO	
CVAEs, n (%)	≥2 CVRFsn = 174	≥3 CVRFsn = 68	≥2 CVRFsn = 156	≥3 CVRFsn = 54	≥2 CVRFsn = 330	≥3 CVRFsn = 122	≥2 CVRFsn = 169	≥3 CVRFsn = 61
Palpitations	0	0	1 (<1)	1 (2)	1 (<1)	1 (<1)	0	0
Supraventricular tachycardia	1 (<1)	0	0	0	1 (<1)	0	0	0
Blood pressure increased	1 (<1)	0	2 (1)	0	3 (<1)	0	0	0
Heart rate increased	0	0	2 (1)	0	2 (<1)	0	1 (<1)	0
ECG QT prolonged	0	0	1 (<1)	0	1 (<1)	0	0	0
Hypertension	3 (2)	2 (3)	1 (<1)	1 (2)	4 (1)	3 (2)	1 (<1)	1 (2)
Hypertensive crisis	0	0	1 (<1)	1 (2)	1 (<1)	1 (<1)	0	0
Hypotension	0	0	1 (<1)	0	1 (<1)	0	0	0
Raynaud’s phenomenon	0	0	1 (<1)	0	1 (<1)	0	0	0

CVAE: cardio- and cerebrovascular adverse event; CVRF: cardiovascular risk factor; PBO: placebo; ECG: electrocardiogram.

**Figure 1.**
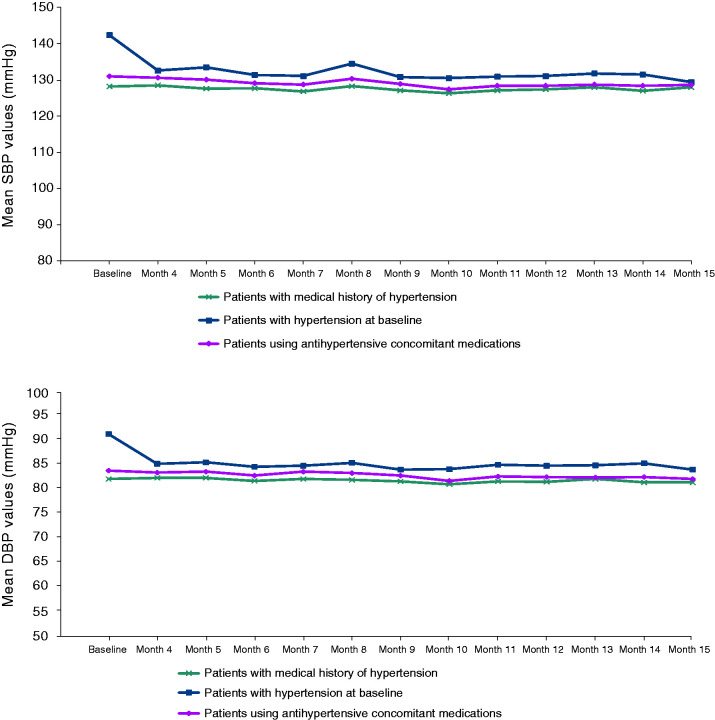
Systolic (a) and diastolic (b) blood pressure values by hypertension status from the long-term safety study. SBP: systolic blood pressure; DPB: diastolic blood pressure.

In a 12-month follow-up study of long-term safety, tolerability, and efficacy of fremanezumab that enrolled patients who completed the HALO CM and EM studies and new patients with migraine (n = 1890) (20), mean systolic and diastolic blood pressure values were consistent or decreased at the end of treatment (up to 15 months, including 3 months in the HALO CM or EM studies and 12 months in the long-term study) compared with baseline values in patients with a medical history of hypertension, patients with hypertension at baseline, and patients using concomitant antihypertensive medications (Figure 1).

### CV safety in patients with baseline and concomitant medication use

The incidence of AEs was similar among patients who used CV medications at baseline compared to the total patient population (quarterly fremanezumab, 66%; monthly fremanezumab, 64%; total fremanezumab, 65%; placebo, 53%; Table 6). Similar to the total patient population, the most commonly reported AEs among patients in this subgroup were injection site related (pain, erythema, induration). Injection site–related AEs, most notably injection-site induration, occurred more frequently in the fremanezumab groups compared to the placebo group in patients receiving CV medications at baseline (injection-site induration: quarterly fremanezumab, 18%; monthly fremanezumab, 24%; total fremanezumab, 21%; placebo, 8%; Table 6). CVAEs occurred at low, similar rates across treatment groups in patients receiving CV medications at baseline, and none of the CVAEs reported occurred in >1 patient (Table 6).

**Table 6.** AEs in patients receiving CV medications at baseline.

AEs, n (%)	Quarterly fremanezumabEM/CM: 675 mg/PBO/PBOn = 87	Monthly fremanezumabEM: 225/225/225 mgCM: 675/225/225 mgn = 91	Total fremanezumabn = 178	PBOn = 102
Patients with ≥1 AE	57 (66)	58 (64)	115 (65)	54 (53)
AEs occurring in >5% of patients in any treatment group				
Injection-site pain	25 (29)	18 (20)	43 (24)	19 (19)
Injection-site erythema	17 (20)	12 (13)	29 (16)	10 (10)
Injection-site induration	16 (18)	22 (24)	38 (21)	8 (8)
Injection-site hemorrhage	2 (2)	4 (4)	6 (3)	2 (2)
Upper respiratory tract infection	3 (3)	6 (7)	9 (5)	5 (5)
Nasopharyngitis	5 (6)	7 (8)	12 (7)	3 (3)
CVAEs				
Tachycardia	0	0	0	1 (<1)
Palpitations	0	1 (1)	1 (<1)	0
Hypertension	1 (1)	1 (1)	2 (1)	0
Hypertensive crisis	0	1 (1)	1 (<1)	0
Hypotension	0	1 (1)	1 (<1)	0

AE: adverse event; CV: cardio- and cerebrovascular; EM: episodic migraine; CM: chronic migraine; PBO: placebo; CVAE: cardio- and cerebrovascular adverse event.

In patients with concomitant triptan use, CVAEs occurred in low, similar proportions across treatment groups (all groups, 2%; Table 7). Increased heart rate and hypertension were the most commonly reported CVAEs in this subgroup; each occurred in <1% of patients in each treatment group. CVAEs were also infrequent in patients without concomitant triptan use (all groups, 3%), and each CVAE occurred in ≤1% of patients (Table S6).

**Table 7.** CVAEs in patients with concomitant triptan use.

CVAEs, n (%)	Quarterly fremanezumabEM/CM: 675 mg/PBO/PBO n = 393	Monthly fremanezumabEM: 225/225/225 mgCM: 675/225/225 mgn = 365	Total fremanezumabn = 758	PBOn = 365
Patients with ≥1 CVAE	6 (2)	7 (2)	13 (2)	6 (2)
Palpitations	1 (<1)	0	1 (<1)	1 (<1)
Tachycardia	0	0	0	1 (<1)
Heart rate increased	1 (<1)	2 (<1)	3 (<1)	1 (<1)
Blood pressure increased	1 (<1)	0	1 (<1)	1 (<1)
ECG PR prolongation	1 (<1)	0	1 (<1)	0
ECG QT prolonged	0	1 (<1)	1 (<1)	0
Hypertension	2 (<1)	1 (<1)	3 (<1)	2 (<1)
Hypotension	0	1 (<1)	1 (<1)	0
Peripheral coldness	0	1 (<1)	1 (<1)	0
Raynaud’s phenomenon	0	1 (<1)	1 (<1)	0

CVAE: cardio- and cerebrovascular adverse event; EM: episodic migraine; CM: chronic migraine; PBO: placebo; ECG: electrocardiogram.

## Discussion

This pooled analysis showed that fremanezumab was safe and well tolerated in a cohort of >2800 patients with migraine from three randomized, double-blind, placebo-controlled, phase 3 clinical trials. The studies included patients with EM and CM, as well as patients with previous inadequate response to two to four classes of migraine preventive medications, thus demonstrating safety and tolerability even in patients with difficult-to-treat migraine. Among patients with migraine, AEs such as weight gain, memory loss, and depression, as well as lack of efficacy, are the most common reasons for discontinuation of migraine preventive treatment (5,22). One longitudinal study found that 75% of patients with CM using oral migraine preventive treatments discontinued treatment by 6 months, and an additional 10% of patients discontinued treatment by 12 months (23). In another study, 24% of patients with EM reported discontinuation of ≥1 preventive medication (24). Compliance for onabotulinumtoxinA is also low; a year-long study reported that only 12.6% of patients followed were treated for 12 months with the prescribed number of cycles (25). The safety profile shown for fremanezumab in a large patient population in this analysis, along with the efficacy data from phase 3 clinical trials (17–20), support its potential as an option for patients seeking safe, tolerable, and efficacious migraine-specific preventive medication.

Fremanezumab is one of four CGRP pathway-targeting mAbs approved for the preventive treatment of migraine, which represent a significant advancement in migraine-specific preventive therapy. Galcanezumab, erenumab, and eptinezumab have generally favorable safety profiles, with serious AEs and AEs leading to discontinuation occurring infrequently across treatment groups (6). The long-term safety of fremanezumab, galcanezumab, and erenumab has also been reported over 12 months of treatment (20,26,27). Fremanezumab, galcanezumab, and erenumab are each administered by SC injection, while eptinezumab is administered by intravenous infusion. Injection-site reactions are commonly reported AEs for those CGRP pathway–targeting antibodies administered by SC injection. Injection-site reactions have been cited as reasons for discontinuation in trials of fremanezumab and galcanezumab, albeit infrequently relative to the occurrence of injection-site reactions (28).

In addition to overall safety and tolerability for patients with migraine, the CV safety of CGRP pathway–targeting antibodies has been a central concern due to CGRP’s role in the cardiovascular system. Here, we show that treatment with fremanezumab over 12 weeks has a favorable CV safety profile, even in patients with CV medical history or CVRFs. Fremanezumab was also safe and tolerable in patients receiving CV medications or triptans, and no safety signals were identified across the three studies included in this analysis. The long-term CV safety of fremanezumab has been demonstrated in a 12-month, double-blind, parallel-group study of >1800 patients, in which CVAEs were infrequent and mostly mild to moderate in severity (20). In that study, similar to the safety profile reported here, hypertension was the most commonly reported CVAE. Hypertension only occurred in 2% of patients, and the majority of those events were single blood pressure elevations in patients with a history of hypertension and were assessed as not related to the study drug (20). For patients with hypertension, patients with hypertension at baseline, and patients using concomitant antihypertensive medications, blood pressure values were similar or numerically lower at the end of up to 15 months of treatment than at baseline, suggesting that fremanezumab did not have an adverse impact on blood pressure in those patients. Possible explanations for the numerically lower post-treatment blood pressure values in patients with hypertension include a secondary effect of having fewer migraine days or improved compliance with antihypertensive medications, although this reasoning is speculative and has not been tested. Likewise, all serious CVAEs in the long-term study were considered not related to the study drug (20). These studies demonstrate the short- and long-term CV safety of fremanezumab for patients with migraine, including those with CV clinical features.

CV safety has also been investigated for galcanezumab and erenumab. In a pooled analysis of six phase 2 and 3 trials of erenumab in patients with EM or CM, vascular AEs were infrequent and occurred in similar proportions of patients across treatment groups (29). There was no association between erenumab treatment and vascular events, including in patients with vascular risk factors at baseline (29). Subgroup analyses of patients using migraine-specific medications showed that the incidences of AEs were similar in patients receiving erenumab or placebo, regardless of the use of triptans or ergots (29). Similar to fremanezumab, hypertension was the most commonly reported CVAE in the erenumab trials; however, its incidence was infrequent and similar between erenumab and placebo treatment groups (29).

In the post-marketing setting, a number of reports have detailed the development of hypertension and worsening of pre-existing hypertension following the use of erenumab, leading to an update to the US prescribing information to include a warning regarding this risk (30). Many of the patients had pre-existing hypertension or risk factors for hypertension. There were cases requiring pharmacologic treatment and, in some cases, hospitalization. Hypertension occurred at different times during treatment but was most frequently reported within 7 days of dose administration. In the majority of the cases, the onset or worsening of hypertension was reported after the first dose, and erenumab was discontinued in many of the reported cases (30). A definitive cause for this observation has not been identified, although it is possible that the mechanism of action for erenumab, which involves targeting the CGRP receptor and is unique among the approved CGRP pathway–targeting mAbs, may be a contributing factor. To date, development or worsening of hypertension has not been reported in the post-marketing setting for fremanezumab, galcanezumab, or eptinezumab (16,31,32).

A pooled analysis of three phase 3 trials of up to 6 months of galcanezumab treatment in patients with EM or CM demonstrated a favorable CV safety profile. Galcanezumab treatment was not associated with an increased incidence of CVAEs compared to placebo, nor changes in blood pressure, pulse, or electrocardiogram features compared to placebo (33). The incidence of hypertension was similar between placebo and galcanezumab treatment groups. A subgroup analysis of patients with concomitant triptan use did not reveal increased risk for CVAEs (33). Together, these studies support the overall and CV safety of CGRP pathway–targeting mAbs, even in individuals who may be at an increased risk for adverse CV outcomes due to CV medical history, CVRFs, or CV medication or triptan use. Although these results are encouraging for the treatment outlook for patients with CV clinical features and migraine, patients at high risk for myocardial infarction or ischemic stroke were excluded from this study and caution should be exercised when prescribing mAbs due to the risk of hypersensitivity-associated acute coronary syndromes (34). A long-term observational safety study of fremanezumab with a nested cohort of CV-compromised patients is currently being conducted to further evaluate the real-world overall safety and CV safety of treatment.

## Conclusion

This pooled safety study showed that fremanezumab treatment was generally safe and well tolerated over 12 weeks in patients with EM or CM, including those with difficult-to-treat migraine. Fremanezumab treatment also showed a favorable CV profile, even in individuals with CV medical history, CVRFs, or concomitant CV medication or triptan use. Patients with high risk of myocardial infarction or stroke were excluded from these studies. Our data support fremanezumab as reasonable for preventive migraine therapy in patients with EM or CM regardless of their CV clinical features.

## Key findings

- Fremanezumab was safe and well tolerated in a pooled cohort of >2800 patients with episodic or chronic migraine, including those with difficult-to-treat migraine.
- Fremanezumab has a favorable cardio-/cerebrovascular (CV) safety profile, even in patients with CV medical history and/or CV risk factors.
- Fremanezumab is safe and well tolerated in patients with concomitant use of cardiovascular medications or triptans.

## Supplemental Material

sj-pdf-1-cep-10.1177_03331024221076485 - Supplemental material for Safety and tolerability of fremanezumab in patients with episodic and chronic migraine: a pooled analysis of phase 3 studies Click here for additional data file. Supplemental material, sj-pdf-1-cep-10.1177_03331024221076485 for Safety and tolerability of fremanezumab in patients with episodic and chronic migraine: a pooled analysis of phase 3 studies by Hans Christoph Diener, Peter McAllister, Tim P Jürgens, Yoel Kessler, Xiaoping Ning, Joshua M Cohen, Verena Ramirez Campos, Steve Barash and Stephen D Silberstein in Cephalalgia
