# Supplementary material for: Safety and tolerability of fremanezumab in patients with episodic and chronic migraine: a pooled analysis of phase 3 studies
Source: Cephalalgia. 2022 Mar 25;42(8):769–80. doi: 10.1177/03331024221076485 (PMC9218411; doi:10.1177/03331024221076485)
Supplement: sj-pdf-1-cep-10.1177_03331024221076485 - Supplemental material for Safety and tolerability of fremanezumab in patients with episodic and chronic migraine: a pooled analysis of phase 3 studies [file sj-pdf-1-cep-10.1177_03331024221076485.pdf]

Supplementary Materials

Table S1. Reported CV Medical History[a]

Medical history, n (%)	Quarterly fremanezumab EM/CM: 675 mg/PBO/PBO n = 943	Monthly fremanezumab EM: 225/225/225 mg CM: 675/225/225 mg n = 954	Total fremanezumab n = 1,897	PBO n = 945
**Hypertension**	80 (8)	76 (8)	156 (8)	87 (9)
**Varicose veins**	13 (1)	10 (1)	23 (1)	7 (<1)
**Reynaud's phenomenon**	12 (1)	10 (1)	22 (1)	7 (<1)
**Hot flush**	6 (<1)	11 (1)	17 (<1)	7 (<1)
**Cardiac murmur**	5 (<1)	6 (<1)	11 (<1)	4 (<1)
**Mitral valve prolapse**	9 (<1)	3 (<1)	12 (<1)	2 (<1)
**Hypotension**	6 (<1)	3 (<1)	9 (<1)	3 (<1)
**Palpitations**	3 (<1)	6 (<1)	9 (<1)	2 (<1)
**Cardiac ablation**	4 (<1)	2 (<1)	6 (<1)	5 (<1)
**Supraventricular tachycardia**	3 (<1)	2 (<1)	5 (<1)	5 (<1)
**Right bundle branch block**	4 (<1)	4 (<1)	8 (<1)	1 (<1)
**Bradycardia**	6 (<1)	0	6 (<1)	3 (<1)
**Tachycardia**	3 (<1)	3 (<1)	6 (<1)	3 (<1)
**Sinus bradycardia**	3 (<1)	3 (<1)	6 (<1)	1 (<1)
**Atrial septal defect**	3 (<1)	2 (<1)	5 (<1)	2 (<1)
**Peripheral venous disease**	3 (<1)	2 (<1)	5 (<1)	2 (<1)
**Mitral valve incompetence**	3 (<1)	2 (<1)	5 (<1)	1 (<1)
**Atrial fibrillation**	2 (<1)	2 (<1)	4 (<1)	2 (<1)
**Arrhythmia**	1 (<1)	2 (<1)	3 (<1)	2 (<1)
**First-degree atrioventricular block**	1 (<1)	2 (<1)	3 (<1)	2 (<1)

**Irregular heart rate**	3 (<1)	1 (<1)	4 (<1)	1 (<1)

CV, cardio- and cerebrovascular; EM, episodic migraine; CM, chronic migraine; PBO, placebo.

[a]Medical history reported for ≥5 patients in the total pooled safety population.

Table S2. Reported CVRFs

CVRF, n (%)	Quarterly fremanezumab EM/CM: 675 mg/PBO/PBO n = 943	Monthly fremanezumab EM: 225/225/225 mg CM: 675/225/225 mg n = 954	Total fremanezumab n = 1,897	PBO n = 945
**Hormonal birth control pill use**	167 (18)	176 (18)	343 (18)	178 (19)
**Smoking**	3 (<1)	7 (<1)	10 (<1)	8 (<1)
**Abnormal ECG**	1 (<1)	0	1 (<1)	1 (<1)
**Albuminuria**	0	1 (<1)	1 (<1)	0
**Atrial fibrillation**	2 (<1)	2 (<1)	4 (1)	2 (<1)
**Diabetes mellitus**	17 (2)	9 (<1)	26 (1)	12 (1)
**Hypertension**	80 (8)	76 (8)	156 (8)	87 (9)
**Impaired glucose tolerance**	5 (<1)	1 (<1)	6 (<1)	4 (<1)
**Lipid metabolism disorders**	77 (8)	69 (7)	146 (8)	70 (7)
**History of CV disease**	153 (16)	145 (15)	298 (16)	142 (15)
**Obesity**	238 (25)	211 (22)	449 (24)	227 (24)
**Sleep apnea**	18 (2)	19 (2)	37 (2)	16 (2)
**Tachycardia**	6 (<1)	4 (<1)	10 (<1)	3 (<1)

CVRF, cardiovascular risk factor; EM, episodic migraine; CM, chronic migraine; PBO, placebo; ECG, electrocardiogram; CV, cardio- and cerebrovascular.

Table S3. Serious AEs and AEs Leading to Discontinuation in the Pooled Safety Population

AEs, n (%)	Quarterly fremanezumab EM/CM: 675 mg/PBO/PBO n = 943	Monthly fremanezumab EM: 225/225/225 mg CM: 675/225/225 mg n = 954	Total fremanezumab n = 1,897	PBO n = 945
**≥1 serious AE**	8 (<1)	12 (1)	20 (1)	17 (2)
**Most common serious AEs (>1 patient in any treatment group)**				
**Drug hypersensitivity**	0	0	0	2 (<1)
**Road traffic accident**	2 (<1)	0	2 (<1)	1 (<1)
**Uterine leiomyoma**	0	0	0	2 (<1)
**AEs leading to discontinuation**	11 (1)	16 (2)	27 (1)	16 (2)
**Most common AEs leading to discontinuation (>1 patient in any treatment group)**				
**Injection-site erythema**	4 (<1)	2 (<1)	6 (<1)	0
**Injection-site rash**	2 (<1)	3 (<1)	5 (<1)	0
**Injection-site pruritus**	2 (<1)	0	2 (<1)	0
**Abdominal pain**	2 (<1)	0	2 (<1)	0
**Diarrhea**	2 (<1)	0	2 (<1)	0
**Drug hypersensitivity**	0	0	0	2 (<1)
**Anxiety**	2 (<1)	0	2 (<1)	0
**Depression**	2 (<1)	0	2 (<1)	0

AE, adverse event; EM, episodic migraine; CM, chronic migraine; PBO, placebo.

Table S4. CVAEs in Patients Without CV Medical History

CVAEs, n (%)	Quarterly fremanezumab EM/CM: 675 mg/PBO/PBO n = 776	Monthly fremanezumab EM: 225/225/225 mg CM: 675/225/225 mg n = 796	Total fremanezumab n = 1,572	PBO n = 792
**≥1 CVAE**	15 (2)	13 (2)	28 (2)	17 (2)
**AEs with occurrence ≥1 patient in any treatment/dose group**				
**Hypertension**	5 (<1)	2 (<1)	7 (<1)	4 (<1)
**Palpitations**	2 (<1)	1 (<1)	3 (<1)	3 (<1)
**Increased blood pressure**	1 (<1)	1 (<1)	2 (<1)	2 (<1)
**Increased heart rate**	1 (<1)	1 (<1)	2 (<1)	2 (<1)
**Hematoma[a]**	2 (<1)	0	2 (<1)	0
**Hot flush**	0	1 (<1)	1 (<1)	2 (<1)
**Tachycardia**	0	1 (<1)	1 (<1)	1 (<1)
**Decreased blood pressure**	1 (<1)	0	1 (<1)	0
**ECG PR prolongation**	1 (<1)	0	1 (<1)	0
**ECG T wave inversion**	0	1 (<1)	1 (<1)	0
**ECG change**	0	0	0	1 (<1)
**Peripheral venous disease**	1 (<1)	1 (<1)	2 (<1)	0
**Hypotension**	0	1 (<1)	1 (<1)	1 (<1)
**Pallor**	1 (<1)	0	1 (<1)	0
**Peripheral coldness**	0	1 (<1)	1 (<1)	1 (<1)
**Superficial vein prominence**	0	1 (<1)	1 (<1)	0
**Temporal arteritis**	0	1 (<1)	1 (<1)	0
**Atrial fibrillation**	0	0	0	1 (<1)

CVAE, cardiovascular adverse event; CV, cardio- and cerebrovascular; AE, adverse event; EM, episodic migraine; CM, chronic migraine; PBO, placebo; ECG, electrocardiogram.

Table S5. CVAEs in Patients With ≥2 CVRFs With or Without CV Medical History

	Quarterly fremanezumab		Monthly fremanezumab		Total fremanezumab		PBO	
**CVAEs, n (%)**	**With CV history n = 112**	**Without CV history n = 62**	**With CV history n = 106**	**Without CV history n = 50**	**With CV history n = 218**	**Without CV history n = 112**	**With CV history n = 112**	**Without CV history n = 57**
**Patients with ≥1 CVAE**	4 (4)	1 (2)	8 (8)	2 (4)	12 (6)	3 (3)	3 (3)	2 (4)
**Palpitations**	0	0	1 (<1)	0	1 (<1)	0	0	0
**Supraventricular tachycardia**	1 (<1)	0	0	0	1 (<1)	0	0	0
**Blood pressure increased**	0	1 (2)	1 (<1)	1 (2)	1 (<1)	2 (2)	0	0
**ECG QT prolonged**	0	0	1 (<1)	0	1 (<1)	0	0	0
**Heart rate increased**	0	0	1 (<1)	1 (2)	1 (<1)	1 (<1)	0	1 (2)
**Hypertension**	3 (3)	0	1 (<1)	0	4 (2)	0	1 (<1)	0
**Hypertensive crisis**	0	0	1 (<1)	0	1 (<1)	0	0	0
**Hypotension**	0	0	1 (<1)	0	1 (<1)	0	0	0
**Raynaud's phenomenon**	0	0	1 (<1)	0	1 (<1)	0	0	0

CVAE, cardiovascular adverse event; CVRF, cardiovascular risk factor; CV, cardio- and cerebrovascular; ECG, electrocardiogram.

Table S6. CVAEs in Patients Without Concomitant Triptan Use

CVAEs, n (%)	Quarterly fremanezumab EM/CM: 675 mg/PBO/PBO n = 550	Monthly fremanezumab EM: 225/225/225 mg CM: 675/225/225 mg n = 589	Total fremanezumab n = 1,139	PBO n = 580
**Patients with ≥1 CVAE**	15 (3)	16 (3)	31 (3)	15 (3)
**Palpitations**	1 (<1)	3 (<1)	4 (<1)	3 (<1)
**Atrial fibrillation**	0	1 (<1)	1 (<1)	1 (<1)
**Supraventricular tachycardia**	1 (<1)	0	1 (<1)	0
**Tachycardia**	0	1 (<1)	1 (<1)	1 (<1)
**Bradycardia**	0	0	0	1 (<1)
**Blood pressure increased**	0	2 (<1)	2 (<1)	1 (<1)
**Blood pressure decreased**	1 (<1)	0	1 (<1)	0
**ECG T-wave inversion**	0	1 (<1)	1 (<1)	0
**ECG change**	0	0	0	1 (<1)
**Heart rate increased**	0	0	0	1 (<1)
**Hypertension**	7 (1)	2 (<1)	9 (<1)	3 (<1)
**Hematoma**	2 (<1)	0	2 (<1)	0
**Hot flush**	1 (<1)	1 (<1)	2 (<1)	2 (<1)
**Peripheral venous disease**	1 (<1)	1 (<1)	2 (<1)	0
**Hypertensive crisis**	0	1 (<1)	1 (<1)	0
**Hypotension**	0	1 (<1)	1 (<1)	1 (<1)
**Pallor**	1 (<1)	0	1 (<1)	0
**Superficial vein prominence**	0	1 (<1)	1 (<1)	0

**Temporal arteritis**	0	1 (<1)	1 (<1)	0
**Peripheral coldness**	0	0	0	1 (<1)

CVAE, cardiovascular adverse event; EM, episodic migraine; CM, chronic migraine; PBO, placebo; ECG, electrocardiogram.
